# Early Neonatal Fosdenopterin Treatment for Molybdenum Cofactor Deficiency Type A: New Insights into Its Natural History and Potential Role for Fetal Therapy

**DOI:** 10.3390/jcm14103561

**Published:** 2025-05-20

**Authors:** Adolfo Etchegaray, Darrah Haffner, Stephanie M. Cruz, Oluseyi Ogunleye, Jason Xia, Amy Schlegel, Oluyinka O. Olutoye, Bimal P. Chaudhari

**Affiliations:** 1 The Fetal Center, Nationwide Children’s Hospital, Columbus, OH 43103, USA; stephanie.cruz@nationwidechildrens.org (S.M.C.); oluseyi.ogunleye@nationwidechildrens.org (O.O.); jxia1@montefiore.org (J.X.); oolutoye@nationwidechildrens.org (O.O.O.); 2 Department of Pediatrics, Division of Pediatric Neurology, Nationwide Children’s Hospital, Columbus, OH 43103, USA; darrah.haffner@nationwidechildrens.org; 3 Department of Pediatric Surgery, Nationwide Children’s Hospital, Columbus, OH 43103, USA; 4 Department of Pediatrics, Division of Neonatology, Nationwide Children’s Hospital, Columbus, OH 43103, USA; amy.schlegel@nationwidechildrens.org; 5 Institute for Genomic Medicine, Nationwide Children’s Hospital, Columbus, OH 43103, USA; bimal.chaudhari@nationwidechildrens.org

**Keywords:** molybdenum cofactor deficiency, fosdenopterin, prenatal diagnosis, fetal therapy, neonatal encephalopathy

## Abstract

**Introduction:** Molybdenum cofactor deficiency (MoCD) is a rare, lethal disorder characterized by early-onset encephalopathy and seizures. In 2021, fosdenopterin (Nulibry^TM^) became the first FDA-approved treatment for MoCD type A (MoCD-A). **Case Presentation:** A G3P2 woman with a prior affected child underwent prenatal diagnosis of MoCD-A at 16 weeks via amniocentesis. Fetal Magnetic Resonance Imaging (MRI) at 22 weeks was normal but showed a mega cisterna magna by 28 weeks. Concerns of ongoing brain damage led to a cesarean section at 32 weeks 6 days estimated gestational age (EGA). Intravenous fosdenopterin was administered within 10 min of birth. Seizures started around 12 h and escalated to status epilepticus by 24 h but resolved by 60 h with treatment. Early MRI demonstrated acute injury without chronic changes. The infant was discharged on day 37 and diagnosed with spastic quadriplegic cerebral palsy at 6 months, with cognition relatively spared. At 24 months, the child remains seizure-free with moderate motor impairment. **Conclusions:** This case highlights that brain injury in MoCD-A may commence in utero during the second trimester. Early delivery combined with immediate neonatal fosdenopterin treatment controlled seizures and halted progression, but residual injury suggests that prenatal interventions are necessary to optimize outcomes.

## 1. Introduction

Molybdenum cofactor deficiency (MoCD) is a group of rare, lethal, autosomal recessive disorders characterized by severe defects in sulfite metabolism. Typical clinical manifestations include facial dysmorphism, intractable epilepsy, excessive irritability, jitteriness, altered muscle tone, feeding difficulties, and autonomic dysfunction [1]. Individuals present with early-onset (severe) or late-onset (mild) phenotypes. Early-onset MoCD is characterized by severe encephalopathy and seizures in the first days of life, similar to the presentation of neonatal hypoxic–ischemic encephalopathy. Neonates may additionally display apnea, poor feeding, limb hypertonia, axial hypotonia, and heightened startle reactions [2]. Early mortality is common. An atypical neonatal presentation mimicking early neonatal sepsis with stridor, respiratory distress, and metabolic acidosis has recently been described [3]. Late-onset MoCD is associated with milder phenotypes and is characterized by episodic neurologic decompensation throughout the lifetime [1]. Beyond the neonatal period, disease progression leads to intractable epilepsy with multiple seizure types and the development of severe dystonic spastic quadriplegia [1,4]. Ocular abnormalities include nystagmus, enophthalmos, spherophakia, colobomas, and lens displacement [5]. Microcephaly, along with other minor dysmorphic facial characteristics, is common [6].

A diagnosis of MoCD is suggested by elevated urinary sulfite and low plasma/urinary uric acid [7,8]. Diagnosis is confirmed by genetic testing, demonstrating biallelic pathogenic variants in molybdenum cofactor biosynthesis protein 1 (MOCS1), molybdenum cofactor biosynthesis protein 2 (MOCS2), molybdenum cofactor biosynthesis protein 3 (MOCS3), or gephyrin (GPHN) [4]. New evidence supporting prenatal detection of early neurological insults to the fetal brain has also been previously described in published reports on fetal MRIs during the third trimester of pregnancy of affected individuals [9,10,11].

**Figure 1:**
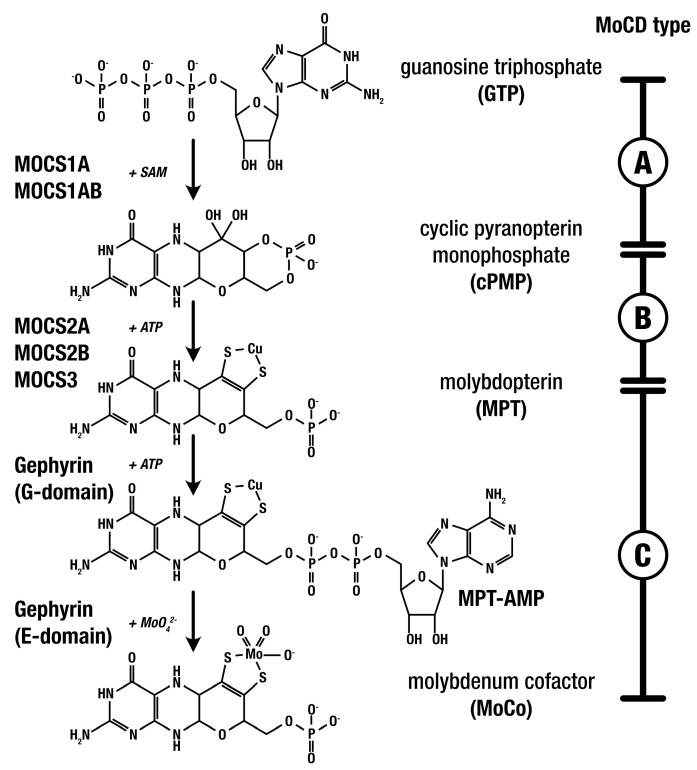
**MoCo biosynthesis:** gene products, MoCD types, and MoCo-dependent enzymes.

The biosynthesis of the molybdenum cofactor (MoCo) from guanosine triphosphate (GTP) involves a critical three-step metabolic pathway (shown in Figure 1). Defects in any of the three steps of MoCo biosynthesis impair its production, reducing the activity of the four MoCo-dependent human enzymes and leading to the respective MoCD subtypes MoCD-A, B, and C [8]. These enzymes are involved in the detoxification and catabolic pathways of sulfur-containing amino acids (such as methionine, homocysteine, cysteine, and taurine) as well as purines (including hypoxanthine and xanthine). The accumulation of intracellular sulfite due to MoCo deficiency leads to increased reactive species production, which in turn has been linked to various detrimental effects, including bioenergetic impairment, mitochondrial permeability transition, and lipid peroxidation [12].

MoCD-A, caused by defects in *MOCS1*, is currently the only type of MoCD with an established medication for treatment [4]. In January 2021, the Food and Drug Administration (FDA) approved the use of fosdenopterin (cyclic pyranopterin monophosphate, cPMP) for the treatment of MoCD-A [13]. Fosdenopterin (Nulibry^TM^, Sentynl Therapeutics, Inc., San Diego, CA, USA) must be administered daily via IV infusion, with treatment initiation early in life for maximum therapeutic benefit.

We present here a case of MoCD-A with early fetal diagnosis and preterm delivery coupled with immediate neonatal treatment with fosdenopterin. We subsequently provide new insights into the intrauterine natural history and future directions for potential prenatal therapies.

## 2. Case Report

**Figure 2:**
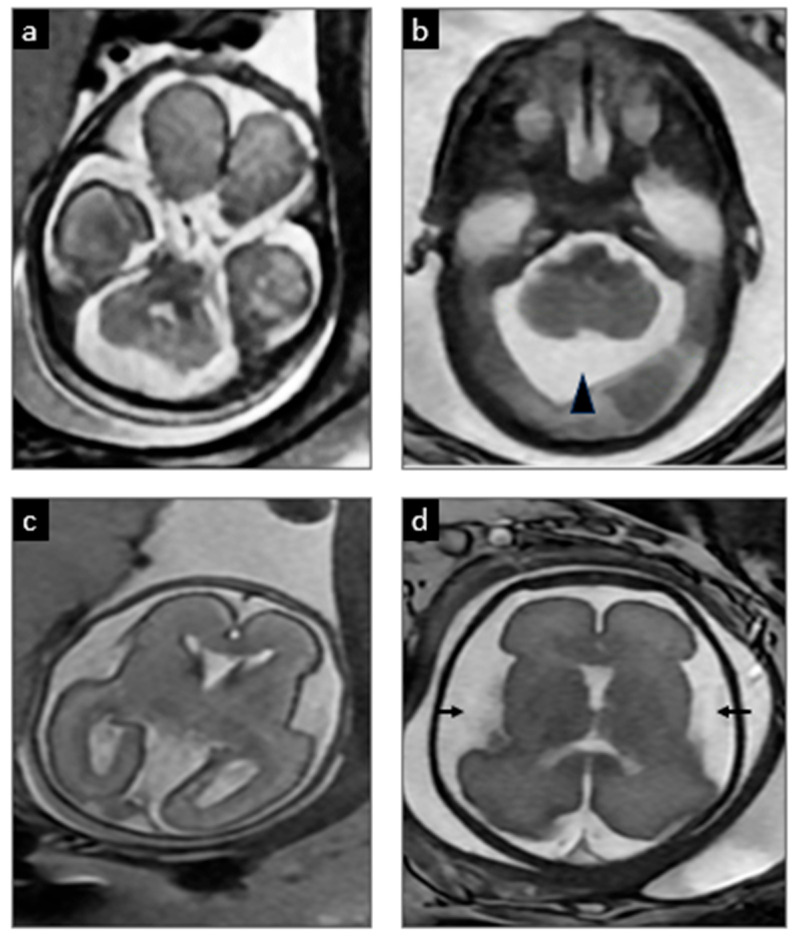
**Serial prenatal MRIs.** At 22 weeks 5 days gestation (**a**,**c**), the initial MRI showed normal development. However, at 28 weeks 5 days (**b**,**d**), there was evidence of a developing mega cisterna magna (arrowhead) and increased extra-axial spaces (arrows), which are common in MoCD and suggested emerging injury.

A 32-year-old pregnant woman, G3P2002, with a history of a previous child affected by MoCD-A, was referred after an amniocentesis at 16 weeks confirmed the presence of the known familial variant [homozygous frameshift truncating variant c.776delA p.(Lys259ArgfsTer10) in MOCS1], concerning for MoCD-A. A baseline MRI obtained at 22 weeks’ gestation demonstrated normal findings with no signs of structural brain injury (shown in Figure 2a,c). A subsequent MRI at 28 weeks revealed a mega cisterna magna and increased extra-axial spaces when compared to the prior study, but no evidence of cerebral white-matter encephalomalacia or edema (shown in Figure 2b,d). While no clear signs of severe brain injury were evident, the subtle changes in the fetal MRI raised suspicions of early neuronal damage. Extensive counseling was provided, discussing the potential benefits of elective preterm birth to reduce fetal brain exposure to neurotoxic metabolites, combined with early initiation of fosdenopterin treatment. These benefits were carefully weighed against the inherent risks of prematurity and the limited data regarding how prematurity might influence the course of the disease, including the possibility of increased susceptibility to injury, particularly in the context of early postnatal therapy. Ultimately, the patient opted for an elective cesarean section at 32 weeks with immediate postnatal treatment. One day prior to the planned delivery, the patient was admitted due to decreased fetal movement and non-reassuring antenatal testing, culminating in a cesarean delivery at 32 weeks and 5 days.

The baby boy’s birth weight was 2060 g. Apgar scores were 6 and 7 at 1 and 5 min, respectively. He was noted to have poor tone and intermittent respiratory effort, requiring Positive Pressure Ventilation (PPV) settings of 20/5. The patient quickly transitioned to Continuous Positive Airway Pressure (CPAP) with settings of a Positive End-Expiratory Pressure (PEEP) of 8 and fraction of inspired oxygen (FiO2) of 21%. The patient had been weaned to room air within the first 10 days of life.

**Figure 3:**
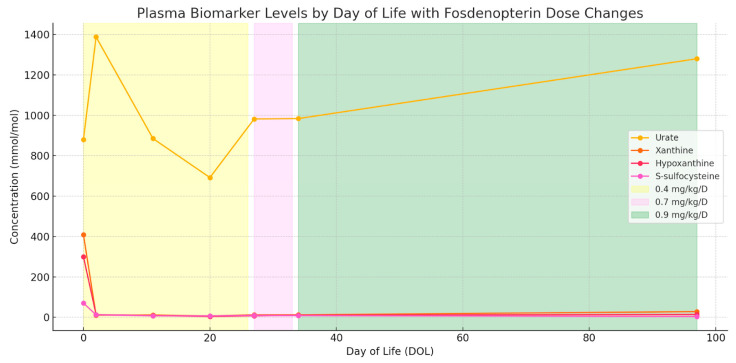
Plasma biomarker trends during fosdenopterin treatment of MoCD-A, aligned to day of life. Concentrations of urate, xanthine, hypoxanthine, and S-sulfocysteine are plotted from day 1 of treatment through 3.5 months of follow-up. Shaded regions indicate fosdenopterin dosing intervals: 0.4 mg/kg/day (yellow), 0.7 mg/kg/day (violet), and 0.9 mg/kg/day (green). Rapid reduction and sustained suppression of toxic metabolites (xanthine, hypoxanthine, and S-sulfocysteine) were observed, while urate remained within the normal reference range throughout treatment.

Initial fosdenopterin infusion was administered within 10 min of delivery, after which the patient was transferred to the NICU for continued care of both MoCD-A and prematurity. Treatment with fosdenopterin followed FDA recommendations for premature children, starting with an initial dose of 0.4 mg/kg/day during the first month, adjusted to 0.7 mg/kg/day until day of life (DOL) 84, and subsequently increased to 0.9 mg/kg/day (Figure 3).

**Figure 4:**
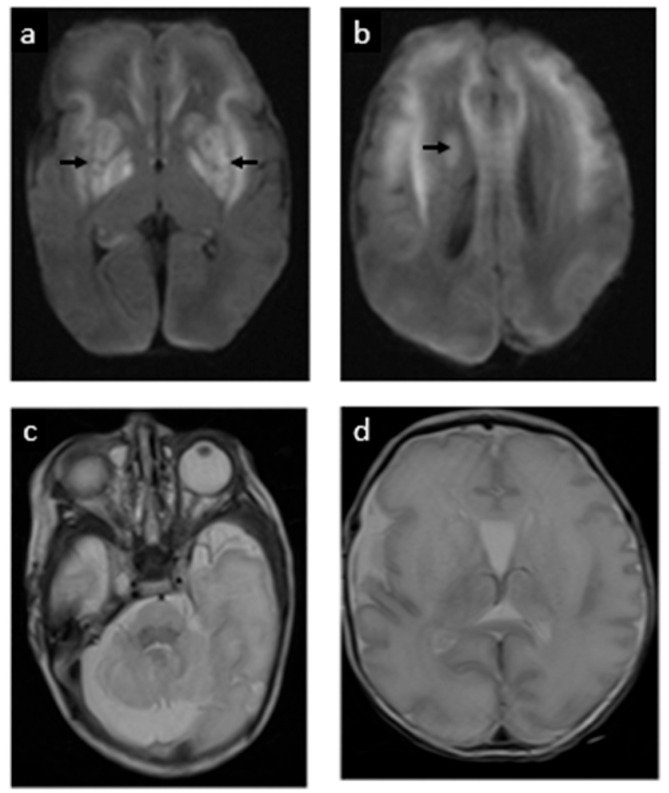
Postnatal MRI at 33 weeks 3 days gestational age equivalent (day 5 postnatally): postnatal MRI revealed acute injury to the basal ganglia, shown as extensive restricted diffusion/cytotoxic edema (white areas in images (**a,b**), signaled with arrows). (**c**) The cerebellum has a normal appearance. (**d**) Simplification of supratentorial gyral pattern, a reflection of prematurity.

**Figure 5:**
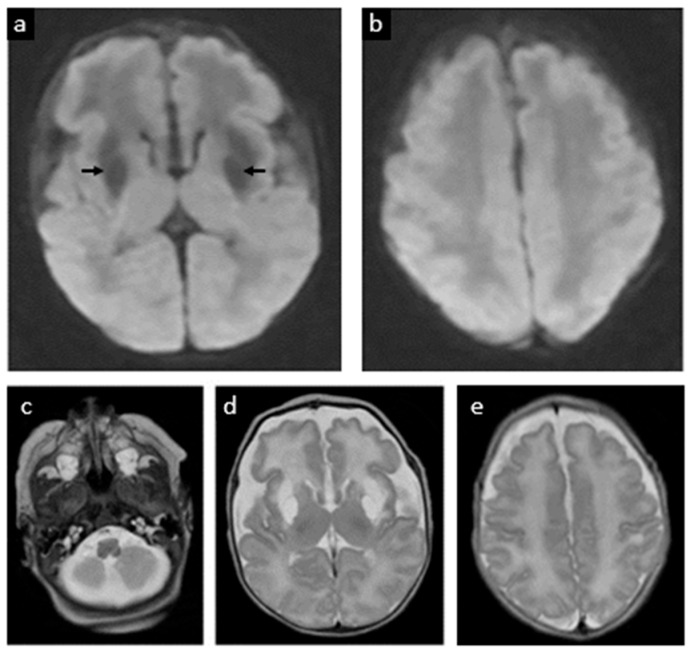
**Follow-up postnatal MRI at 37 weeks 4 days gestational age equivalent (week 5 postnatally).** (**a**,**b**) Previous areas of ischemia have evolved to cystic encephalomalacia in the bilateral basal ganglia and frontal perisylvian white matter (dark areas in image a signaled with arrows). (**c**) The brainstem and cerebellum appear normal. Large CSF spaces in the posterior fossa. (**d**) Volume loss of the frontal lobes with abnormal T2 hyperintense signal of the white matter. (**e**) Cerebral sulcation has progressed. No new parenchymal abnormalities. Myelination pattern is overall normal.

Electrographic seizures were first noted at 12 h of life. These were refractory to phenobarbital and fosphenytoin, ultimately requiring continuous midazolam infusion. Seizures resolved on DOL 2. The physical exam at that time also indicated encephalopathy, but no focal neurologic deficits. On DOL 5, a brain MRI showed extensive restricted diffusion in the bilateral basal ganglia, frontal white matter, internal capsules, and brainstem corticospinal tracts, as well as the callosal genu and hippocampi without cystic changes, suggestive of acute but no chronic MoCD-related neurological injury (shown in Figure 4). A mega cisterna magna was also confirmed. Repeat MRI at term corrected age revealed cystic encephalomalacia in the bilateral basal ganglia and frontal perisylvian white matter, correlated to prior areas of restricted diffusion. There were no new areas of injury (shown in Figure 5).

The infant initially required CPAP for respiratory support and was transitioned to room air on DOL 11. Human milk feeds were started on DOL 2, but the infant developed necrotizing enterocolitis (NEC) on DOL 6 based on clinical examination of abdominal distension and radiologic findings of pneumatosis. A 7-day course of empirical antibiotics, consisting of ampicillin/sulbactam and gentamicin, was completed without complications. Regular monitoring included weekly purine and pyrimidine panels, as well as continuous assessment of urinary uric acid levels throughout the hospitalization. The patient received protein-restricted Total Parenteral Nutrition (TPN) fortified with thiamine and steadily advanced to full cysteine-restricted, fortified feeds after completion of treatment for NEC. At discharge at 38 weeks corrected age, a neurological exam was normal and a repeat electroencephalogram (EEG) demonstrated no abnormalities.

The child continues under treatment with fosdenopterin, with close neurodevelopmental follow-up. He has had no seizure recurrence beyond the neonatal period. The child does have prominent motor delays and was diagnosed with cerebral palsy at age 7 months, with significant dystonia affecting his upper more than his lower extremities. While he has global delays, cognition and receptive and expressive communication are relatively spared. At 24 months corrected age, the child is sitting unsupported, has a few oral words, and is using an eye gaze device. This is in contrast to his older brother, who at six years of age is unable to sit independently, support his own head, or vocalize beyond cooing, and has intractable epilepsy.

## 3. Discussion

### 3.1. Diagnosis and Natural History

Depending on the gestational age, prenatal diagnosis of MoCD can be accomplished by an assay of the enzyme sulfite oxidase in a chorionic villus specimen or by an increase in S-sulfocysteine (SSC) in the amniotic fluid, although this is rarely performed. In cases where the specific gene is known, such as in instances of a prior affected child, targeted mutation analysis of these genes is performed to confirm the diagnosis [14].

The first description of prenatal brain damage in MoCD comes from a case report of a patient with diffuse brain damage manifested as multiple subcortical cavities, ventriculomegaly, dysgenesis of the corpus callosum, and a hypoplastic cerebellum with an enlarged cisterna magna in a 35-week ultrasound. MRI subsequently showed brain atrophy, and multicystic encephalomalacia with hypoplastic vermis and cerebellum [9]. Lubout et al. [10] later described two cases of MoCD monitored prenatally by serial fetal MRIs starting at 20 and 32 weeks, respectively. The first case showed prominent lateral ventricles and a slight increase in signal intensity of the cerebral white matter at 36 weeks. In the second case, a mega cisterna magna was observed at 32 weeks, and later at 36 weeks, prominent lateral ventricles and a slight increase in cerebral white matter signal intensity were noted. Fosdenopterin or cPMP substitution therapy was started 4 and 5 h after birth, respectively, but both infants showed significant neurodevelopmental delay. Mega cisterna magna, which is non-specific, is the most consistent prenatal finding in the small series of prenatal cases available in the literature.

### 3.2. Treatment Approaches

In MoCD-B and -C, no treatments have been developed due to the short half-life of these substrates. MoCD-A stands out as the only subtype with a recognized treatment strategy, involving the daily administration of either bacteria-derived or chemically synthesized cPMP. Substrate replacement therapy provides an exogenous source of cPMP, which undergoes conversion into molybdopterin. This, in turn, transforms into molybdenum cofactor, essential for activating molybdenum-dependent enzymes. Upon treatment initiation, urinary levels of SSC, thiosulfate, xanthine, and uric acid can be monitored, exhibiting a decline with appropriate cPMP administration. Patients typically experience minimal side effects, and neurodegeneration is drastically slowed. The first case of intravenous replacement therapy with cPMP was reported in 2010 by Veldman et al. [15], in which a girl who was diagnosed with MoCD-A at the age of 6 days received intravenous cPMP from DOL 36. Within 2 weeks, all urinary markers of sulfite oxidase and xanthine oxidase deficiency returned to almost normal and remained stable thereafter. Clinically, the infant displayed increased alertness, and seizures ceased within the initial two weeks of treatment. In 2015, a cohort of 11 neonates treated with cPMP demonstrated a reduction in disease biomarker levels to normal in all cases, accompanied by symptom improvement, with three patients achieving near-normal development [16]. Notably, in 2021, the FDA approved a synthetic cPMP known as fosdenopterin (Nulibry^TM^) [13].

Additional supportive treatments for MoCD, including dietary restriction of sulfur-containing amino acids [17], have shown efficacy in delaying neurodegeneration, particularly in patients with milder forms of MoCD. Other potential treatment modalities under consideration encompass N-methyl-D-aspartate (NMDA) receptor antagonists [18], sulfite scavenging, and ferroptosis inhibition.

### 3.3. Review of the Literature: Neonatal Treatment with Fosdenopterin

We conducted a comprehensive review of the medical literature on neonatal cases of MOCD-A treated with fosdenopterin. PubMed (http://www.ncbi.nlm.nih.gov/pubmed accessed on 10 January 2024) and the Cochrane Library (https://www.cochranelibrary.com, URL accessed on 10 January 2024), using the search terms [molybdenum cofactor deficiency OR MOCS1] AND [fosdenopterin OR cyclic pyranopterin monophosphate] [human].

From an initial search yielding 23 results, 5 peer-reviewed publications met our inclusion criteria, focusing on cases where treatment commenced within the neonatal period. These included two single case reports [19,20] and several small case series covering both neonatal and postneonatal stages. Table 1 compiles all the published, peer-reviewed case reports or series of genetically confirmed molybdenum cofactor deficiency type A (MoCD-A) treated with fosdenopterin (cPMP) during the neonatal period [16,20,21].

### 3.4. Future Directions

As with many intoxicating inborn errors of metabolism, the initiation of therapeutic efforts prior to end-organ impairment is crucial, as disease-related neurological injury is largely irreversible. In early cases of MoCD-A treated with E. coli-derived cPMP, despite initial promising metabolic improvements, significant neurological impairment was still observed. However, this was due to irreversible brain injury sustained either prenatally or shortly after birth and prior to the initiation or full effect of treatment, rather than ongoing disease progression during the treatment period. Typically, these treatments were initiated after symptoms had already developed. In a cohort study by Schwahn et al. [16], the age at onset of treatment was between 0 and 68 days, with a median of 7 days. The two patients in which treatment started at day of life 0, before severe encephalopathy, experienced rapid improvement in symptoms, with no clinically significant disability on long-term follow-up. MRI scans 2–4 years into treatment showed normal or mild atrophy with no irreversible changes. However, these patients still experienced speech delay with a low normal range for cognitive function, and long-term outcomes are still unknown.

This case contributes to the literature by providing further imaging evidence of prenatal anatomical brain injury in MoCD-A, identified at 28 weeks gestation, consistent with previous reports indicating such injuries can occur as early as 21 weeks gestation [21]. Additionally, this case represents the earliest initiation of neonatal fosdenopterin treatment reported, starting within 10 min after birth at 32 weeks and 6 days corrected gestational age. Although the infant initially experienced seizures and imaging abnormalities indicative of early brain injury, seizures ceased shortly after treatment initiation, clinical examination improved, and subsequent imaging revealed no further progression, suggesting a potentially favorable outcome.

The findings of this case indicate that the onset of brain damage in individuals with MoCD may occur earlier than initially believed. They also suggest that, although elective premature birth coupled with immediate neonatal treatment may potentially improve neurologic outcomes by shortening the exposure to toxic sulfites, this neuroprotection is insufficient, as substantial brain damage still occurred. It is suspected that in this case, the toxic insult had already become significant when the mother presented with decreased fetal movement the day prior to the scheduled caesarian section. However, it is important to recognize that prematurity carries inherent risks, and escalating levels of prematurity are also associated with a higher burden of neurologic morbidity and increased mortality risk. Consequently, there is a compelling rationale to investigate future prenatal interventions aimed at neutralizing damage during the early stages of gestation rather than further pursuit of iatrogenic preterm delivery in hopes of instituting treatment prior to brain injury.

The potential fetal therapy options for molybdenum cofactor deficiency type A (MoCD-A) would include currently available approaches, such as the administration of fosdenopterin through umbilical cord infusions, to more definitive therapeutic strategies like gene therapy. Regarding prenatal fosdenopterin infusions, while previous concerns focused primarily on the short half-life of fosdenopterin (1.2 to 1.7 h) necessitating daily administration, which poses an unacceptable cumulative risk of procedure-related prematurity, recent pharmacodynamic data indicate prolonged biological effects extending beyond initial expectations. Clinical and metabolic benefits, including sustained activity of molybdenum cofactor-dependent enzymes, persisted for at least one week following withdrawal, according to a recent publication [22], suggesting a biological half-life for these enzymatic activities of approximately three days. This evidence supports the feasibility of less frequent dosing intervals, potentially reducing the cumulative procedural risks associated with daily administration. Ideally, fosdenopterin could be administered at intervals of once every 1–2 weeks, significantly enhancing the practicality and safety of prenatal therapy, especially when initiating treatment before 28 weeks of gestation. Nevertheless, pharmacokinetic studies in fetuses are still lacking, and the optimal gestational age to commence therapy remains uncertain, although recent evidence, including our case, suggests that it should probably be before 26 to 28 weeks.

Postnatal gene therapy using an adeno-associated virus (AAV) vector expressing the MOCS1 gene showed promising results, extending survival and rescuing the phenotype in MOCS1-deficient mice [23]. To date, no cases of fetal gene therapy have been reported in humans for any single-gene disorder. However, fetal stem cell transplantation has been utilized in various genetic disorders, including metabolic conditions like acute neuronopathic (type II) Gaucher disease, Hurler syndrome, and Niemann–Pick type A [24]. Additionally, ongoing clinical trials are exploring the treatment of α-thalassemia major (NCT02986698) [25] and osteogenesis imperfecta (NCT03706482) [26] with hematopoietic and mesenchymal stem cells, respectively. The EVERREST trial (NCT02097667) [27] stands out as one of the main candidates to become the first fetal gene therapy trial. At present, it is actively collecting data to identify ultrasound and/or biochemical prognostic markers in pregnancies affected by severe early-onset Fetal Growth Restriction (FGR). This information is intended to serve as a comparison with a future group slated to undergo gene therapy utilizing maternal vascular endothelial growth factor (VEGF), as part of the phase I/IIa trial.

One of the main limitations of fetal therapy for metabolic diseases is the recognition of specific fetal phenotypes. A minority of these conditions may manifest prenatally as fetal hydrops [28,29], but in such cases, it is likely too late to reverse the organic damage. The only way to recognize these cases in a timely manner, especially when there is no family history of a previously affected sibling, may be through the implementation of preconception population-based carrier screening programs. Alternatively, prenatal screening programs during the first or early second trimester could enable parents to make decisions about potential early therapies. For such a screening program to be acceptable and cost-effective, it would probably need to be conducted non-invasively (akin to cell-free-DNA screening for fetal aneuploidy). Moreover, it would also need to offer effective therapies with significant long-term fetal benefits and acceptable risks for both the fetus and the mother.

This case highlights that neurologic injury in molybdenum cofactor deficiency type A may begin as early as the second trimester, underscoring a narrow and critical window for effective neuroprotection. While early delivery and immediate neonatal treatment with fosdenopterin successfully controlled seizure activity and prevented further injury, the presence of pre-existing brain damage resulted in significant long-term motor impairment. These findings underscore the limitations of postnatal intervention alone and point to a pressing need to investigate prenatal therapies capable of neutralizing neurotoxic damage before irreversible injury occurs. Ultimately, this case illustrates both the promise and the current challenges in optimizing outcomes for fetuses affected by MoCD-A. Nonetheless, as a single case report, the generalizability of these findings is limited, and further research is needed to validate the benefits and risks of early delivery and immediate neonatal treatment in a broader population of patients with MoCD-A.

## Figures and Tables

**Table 1:** Neonatal MoCD type A cases treated with fosdenopterin (literature review).

Case	Author (Year)	Prenatal Diagnosis	Prenatal Findings	Day of First Fosdenopterin Dose	Corrected GA at Start	Clinical Outcome
1	Hitzert et al., 2012 [20]	Yes	None reported	Day 0 (4 h after birth)	36 w 3 d	Brief neonatal seizures; mildly delayed cognitive, rest normal neurodevelopment at follow-up (21 months)
2	Schwahn et al., 2015 (Patient 3) [16]	No (family history; not prenatal)	None reported (dysmorphic features at birth)	Day 7	~40 w	Seizure-free, near-normal development long-term (21 months)
3	Schwahn et al., 2015 (Patient 4) [16]	No	None reported (dysmorphic features at birth)	Day 5	~42 w	Marked improvement, but residual neurologic deficits (not fully seizure-free)
4	Schwahn et al., 2015 (Patient 5) [16]	Yes (older sib; prenatal)	None reported (dysmorphic features at birth)	Day 0	~36 w	Seizure-free, normal development by age 3 years
5	Schwahn et al., 2015 (Patient 6) [16]	No (family history; not prenatal)	None reported	Day 4	~39 w	Initial improvement, parents decided to abandon treatment after 111 days and the patient was lost to follow-up
6	Schwahn et al., 2015 (Patient 7) [16]	Yes (older sib; prenatal)	None reported (dysmorphic features at birth)	Day 0	40 w	Seizure-free, near-normal development long-term by age 2 years
7	Schwahn et al., 2015 (Patient 8) [16]	No	None reported	Day 11	~40 w	Transient stabilization, but died in neonatal period (~3 weeks)
8	Schwahn et al., 2015 (Patient 10) [16]	No	None reported	Day 8	~41 w	Rapid clinical decline; died in neonatal period (~11 days old)
9	Schwahn et al., 2015 (Patient 11) [16]	No	None reported	Day 6	~42 w	Survived; treatment stopped after 5 days (irreversible damage)– Severe CP, intractable impairment
10	Lubout et al., 2018 (Patient A) [10]	Yes (via chorionic villus sampling)	Fetal MRI normal until ~36 wk; then mild ventriculomegaly and subtle white-matter T2 hyperintensities observed	Day 0 (~4 h after birth)	36 + 4 w	Neonatal seizures controlled; at 41 mo ~30–35 mo developmental level (Bayley ~16th percentile) with persistent macrocephaly (HC > P98; familial trait)
11	Lubout et al., 2018 (Patient B) [10]	Yes (via amniocentesis at 32 weeks)	Fetal MRI from 32 wk: mega cisterna magna with slightly small cerebellum; by 36 wk mild ventriculomegaly and subtle white-matter T2 hyperintensities	Day 0 (~5 h after birth)	~39–40 wk	Only brief subclinical neonatal seizures (on aEEG) at 41 mo~18–26 mo developmental level (Bayley; cognitive ~5th, motor ~0.5th percentile), with clumsy gait
12	Schwahn et al., 2024 (Patient A) [21]	No	None reported	Day 7	Term	No seizure control. Severe dystonic and spastic quadriplegia. Received cPMP for 7 days before brain MRI results showed widespread severe diffusion restriction and signs of brain necrosis
13	Schwahn et al., 2024 (Patient B) [21]	No	None reported	Day 3	Term	No seizure control. Severe dystonic and spastic quadriplegia. Received cPMP for 8 days before stopping due to lack of benefit
14	Schwahn et al., 2024 (Patient C) [21]	No	None reported	Day 4	Term	No seizure control. Severe dystonic and spastic quadriplegia. Received cPMP for 22 days before stopping due to lack of benefit
15	Schwahn et al., 2024 [19]	No	None reported	Day 4	39 w 2 d	Early brain MRI at 5 days revealed transient edema and signal changes in globi pallidi, resolving by week 6. Long-term neurodevelopment showed mild muscular hypotonia and delayed cognitive milestones
16	Etchegaray et al., 2025 (this case)	Yes (16 w via amniocentesis)	Fetal MRI normal at 22 w; mega cisterna magna at 28 w	Day 0 (within 10 min of birth)	32 w 6 d	Neonatal seizures (resolved by 60 h); discharged D37. Developed dystonic quadriplegic CP by 6 mo (cognition relatively spared); remains seizure-free at 24 mo

## Data Availability

All data generated or analyzed during this study are included in this article. Further inquiries can be directed to the corresponding author.
